# Insomnia and the question of psychomotor agitation as a possible cause for zolpidem abuse

**DOI:** 10.36416/1806-3756/e20250459

**Published:** 2026-03-05

**Authors:** Sivan Mauer, Alexandre Karam Joaquim Mousfi, Geraldo Lorenzi-Filho

**Affiliations:** 1 Department of Psychiatry, Tufts University Medical School, Boston, MA, USA.; 2 Disciplina de Psiquiatria, Faculdade Evangélica Mackenzie do Paraná, Curitiba (PR) Brasil.; 3 Serviço de Psiquiatria, Hospital Universitário Evangélico Mackenzie, Curitiba (PR) Brasil.; 4 Instituto do Coração - InCor - Hospital das Clínicas, Faculdade de Medicina, Universidade de São Paulo - HCFMUSP - São Paulo (SP) Brasil.

For a long time, obstructive sleep apnea (OSA) and insomnia were believed to be two opposing poles. The stereotype of OSA is that of an obese, sleepy middle-aged man. In contrast, insomnia was thought to be the opposite, predominantly affecting women. Studies published in the last decade showed a large overlap between OSA and insomnia, known as comorbid insomnia and sleep apnea. Approximately 30-50% of patients with OSA meet criteria for insomnia, and approximately 30-40% of those with chronic insomnia have OSA.^1^ Comorbid insomnia and sleep apnea is associated with worse outcomes than is OSA or chronic insomnia alone. In clinical practice, poor sleep quality is a common complaint among patients who have been prescribed sleep-inducing drugs, particularly zolpidem, without an in-depth investigation into the origin and characteristics of their insomnia. Insomnia can be classified as difficulty initiating sleep, difficulty maintaining sleep, or early morning awakening, leading to daytime impairments. It is important to emphasize that sleep-inducing drugs are not indicated for patients who have difficulty maintaining sleep or who wake up earlier than desired. In such patients, a systematic investigation of comorbidities, particularly psychiatric disorders and OSA, is also fundamental. 

The industry has always tried to portray zolpidem as a safe drug with a low risk of addiction. For several years there was little control over the prescription of zolpidem in Brazil; however, approximately one year ago there was a change in legislation, with greater control over its dispensation. From 2014 to 2021, there was a 246% increase in so-called “Z-drugs,” which include zolpidem, zopiclone, eszopiclone, and zaleplon.^2^ From 2018 to 2022, there was a 67% increase in sales of zolpidem, according to the Brazilian Health Regulatory Agency.^3^ Zolpidem has a very short half-life (of 1.5-3 h), which is a risk factor for tolerance, an important concept for the development of dependence. Tolerance is a progressive reduction in the intensity of the effect of a substance with repeated use, such that higher doses are needed to achieve the same effect initially obtained with lower doses.^4^ Some studies have shown a downward trend in the prescription of long-acting benzodiazepines.^5^ ^,^ ^6^

The aforementioned data are important because most insomnia cases are secondary to psychiatric and clinical illnesses; in other words, insomnia is a symptom of a primary disease rather than a disease per se.^7^ ^-^ ^9^ It is of note that most patients with insomnia present with psychomotor agitation, which is possibly one of the most common causes of insomnia.^10^ Psychomotor agitation, whether mild or intense, is a central symptom of mood disorders. 

Since the late 19th century, psychomotricity has been recognized as one of the phenomenological cores of mood disorders, representing the meeting point between the body and affectivity. Emil Kraepelin described psychomotor retardation or agitation as cardinal manifestations of manic-depressive illness, giving motricity a central diagnostic value for being more objective than subjective mood variations.^11^ **Karl Jaspers, in his *General Psychopathology* (originally published in 1913), expanded this view by understanding movement as a visible expression of the emotional state, the “speaking body” of affective experience.** ^12^ In the German tradition, authors such as Wernicke, Kleist, and Binswanger associated psychomotor alterations with the disorganization of the “vital rhythm” and internal temporality, anticipating contemporary phenomenological interpretations.^13^ ^,^ ^14^ In contemporary thought, Ghaemi takes up this Kraepelinian legacy, emphasizing that mood disorders are essentially illnesses of energy and vital motor function, and that psychomotor observation remains the most reliable clinical marker of cyclothymia and melancholia, articulating body, time, and affect in a unified dimension of human experience.^15^ For Ghaemi, manic-depressive illness is not merely a series of manic and depressive episodes, but a unified and continuous clinical entity that expresses a fundamental alteration in temperament, energy, and affective motricity. Inspired by Kraepelin, Ghaemi argues that this condition should be understood as a mood disorder in a broad sense, characterized by cyclical fluctuations in vitality-a dysregulation of the “rhythm of psychic life” that affects thought, behavior, and emotion. He criticizes modern diagnostic fragmentation (such as the separation between unipolar depression and bipolar disorder) for reducing the phenomenon to artificial and descriptive categories. For Ghaemi, the Kraepelinian model remains valid because it recognizes the longitudinal and temperamental dimension of the disorder, emphasizing that the core of manic-depressive illness is a disturbance of vital energy rather than of isolated emotion. This view implies a psychiatric paradigm that integrates biology, phenomenology, and life history, recognizing the affective course and style as central to diagnosis and treatment.^15^

Affective temperament can be understood as a chronic, subclinical, and attenuated manifestation of manic-depressive illness, representing the most stable axis of the bipolar spectrum.^16^ ^,^ ^17^ Temperament reflects a persistent psychobiological disposition rooted in the systems that regulate mood, energy, and circadian rhythm. Thus, it is also expressed in the physiological functions of sleep and wakefulness, which constitute the somatic correlate of affective cyclicity. Individuals with hyperthymic (subclinical and chronic manic symptoms) or cyclothymic temperaments frequently exhibit reduced sleep, minimal need for rest, and spontaneous early awakenings, consistent with a baseline state of dopaminergic hyperactivity and accelerated biological rhythms.^18^ On the other hand, those with dysthymic temperaments (subclinical and chronic depressive symptoms) tend to present with hypersomnia, morning fatigue, and nonrestorative sleep, expressing a chronic decrease in vital energy and circadian responsiveness. These variations are not epiphenomena; rather, they are continuous expressions of the same energetic-temporal axis that in extreme forms manifests clinically as mania or melancholy.^19^ Thus, sleep patterns become a biological and phenomenological marker of the existential rhythm of the patient, revealing the continuity between temperament, vulnerability, and the clinical expression of bipolar disorder. 

An understanding of the aforementioned concepts, especially those related to affective temperaments, is particularly important in addressing these conditions, given that improved sleep depends on the regulation of psychomotor activity. Treatment of affective temperaments-particularly cyclothymic and hyperthymic temperaments-may include the use of low, individualized doses of mood stabilizers to modulate affective reactivity and prevent progression to more severe clinical episodes.^20^ ^,^ ^21^ Many patients who experience subtle mood swings, irritability, or chronic variations in energy and sleep belong to the subclinical manic-depressive spectrum and benefit from mild prophylaxis, aimed more at stabilizing rhythm and vital energy than at full symptomatic remission. Drugs such as lithium (below 300 mg/day) and sodium valproate (250-500 mg/day) have been shown to reduce affective instability without inducing emotional blunting and to improve sleep patterns following a decrease in psychomotor agitation.^20^ ^-^ ^23^
